# Use of the OpinionFamily program to improve satisfaction among families of intensive care unit patients

**DOI:** 10.1186/s13054-023-04445-2

**Published:** 2023-05-10

**Authors:** Vincent Labbé, Elise Morawiec, Naïke Bigé, Irma Bourgeon-Ghittori, Keyvan Razazi, Armand Mekontso Dessap, Sophie Tuffet, Alexandra Rousseau, Muriel Fartoukh, the OpinionFamily Group, Aude Gibelin, Guillaume Voiriot, Michel Djibré, Clarisse Blayau, Martin Dres, Maxens Decavèle, Alexandre Demoule, Hafid Ait-Oufella, Bertrand Guidet, Jérémie Joffre

**Affiliations:** 1 Service de Médecine Intensive Réanimation, Département Médico-Universitaire APPROCHES, Centre Hospitalier Universitaire Tenon, Assistance Publique-Hôpitaux de Paris, Sorbonne Université, Paris, France; 2 Service des Soins Intensifs, Hôpital Universitaire de Bruxelles, Université Libre de Bruxelles, Brussels, Belgium; 3 Institut Mondor de Recherche Biomédicale, Groupe de Recherche Clinique CARMAS (Cardiovascular and Respiratory Manifestations of Acute lung injury and Sepsis), Université Paris Est Créteil, Créteil, France; 4 Service de Médecine Intensive Réanimation, Centre Hospitalier Universitaire Pitié-Salpêtrière, Assistance Publique-Hôpitaux de Paris, Sorbonne Université, Paris, France; 5 Service de Médecine Intensive Réanimation, Centre Hospitalier Universitaire Saint-Antoine, Assistance Publique-Hôpitaux de Paris, Sorbonne Université, Paris, France; 6 Département Médico-Universitaire SAPHIRE, Hôpitaux Universitaires Henri Mondor-Albert Chenevier, Assistance Publique-Hôpitaux de Paris, Créteil, France; 7 Service de Médecine Intensive Réanimation, Département Médico-Universitaire Médecine, Hôpitaux Universitaires Henri Mondor-Albert Chenevier, Assistance Publique-Hôpitaux de Paris, Université Paris Est Créteil, Créteil, France; 8 Institut Mondor de recherche biomédicale, Institut national de la santé et de la recherche médicale, Université Paris Est Créteil, Créteil, France; 9 Department of Clinical Pharmacology and Clinical Research Platform Paris-East (URCEST-CRC-CRB), Centre Hospitalier Universitaire Saint-Antoine, Assistance Publique-Hôpitaux de Paris, Sorbonne Université, Paris, France

To the editor

Meeting the needs of families of intensive care unit (ICU) patients has emerged as a major target to reduce the risk of post-traumatic stress disorder [1, 2]. The OpinionFamily program was developed by a 12-member working group including intensivists, nurses, and a sociologist, in partnership with “ChooseMyCompany.com”, a company specializing in satisfaction surveys, to assess family satisfaction anonymously over time, and to provide regular feedback reports to caregivers to facilitate prompt implementation of appropriate improvement interventions. The program combines (i) 24/7 availability of a Critical Care OpinionFamily Survey (CCOFS) on a touch screen in the ICU waiting room for confidential completion by patients’ relatives (Fig. 1A; Additional file 1: Methods S1); (ii) a feedback report sent to each center every 3 months (baseline, periods 1, 2, and 3); and (iii) implementation of report-based interventions at each center (Additional file 1: Methods S2). The CCOFS included six dimensions (Proximity to the patient, Comfort, Availability of caregivers, Trust, Support, and Information; Additional file 1: Table S1) according to validated measures of family satisfaction [1, 3, 4].

**Fig. 1:**
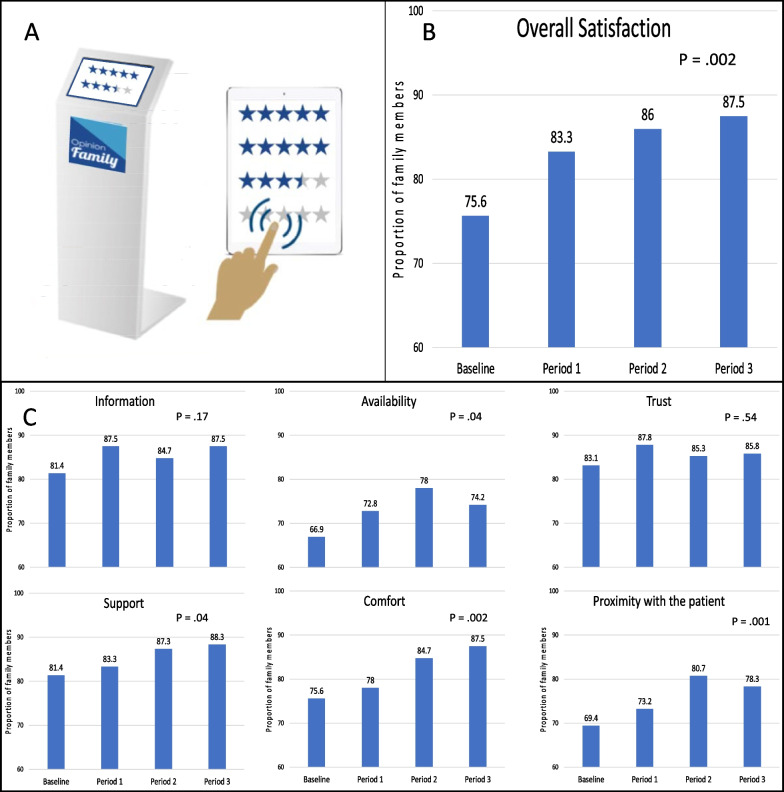
Family Satisfaction Across the Study Periods as assessed using the Critical Care OpinionFamily Survey. **A** The OpinionFamily secure touch screen available 24/7 in the waiting room of the intensive care unit, **B** Overall family satisfaction; **C**, Family satisfaction for each of the six satisfaction-related dimensions. *P* values shown on the figure are for the evolution over time of the proportion of satisfied family members (baseline, *n* = 242; period 1, *n* = 287; Period 2, *n* = 150; Period 3, *n* = 120) using Mantel–Haenszel’s chi-square test for trend

In this prospective study, we evaluated the effectiveness of the OpinionFamily program on family satisfaction between December 2018 and December 2019 in four French university hospital ICUs (characteristics in Additional file 1: Table S2). Our framework for identifying and auditing corrective actions was based on Plan-Do-Study-Act cycles. The primary outcome was the proportion of satisfied family members at each period; secondary outcomes were the proportion of satisfied family members for each dimension at each period. The Comité de Protection des Personnes Ile-de-France5 (N°e-5-16) approved the protocol.

Changes in family satisfaction over time were analyzed using Mantel–Haenszel’s chi-square test for trend, and a mixed generalized linear regression model with a binomial distribution and a logit link function, taking the period as fixed effect and the center as random effect. Odds ratios (OR) with 95% confidence intervals (CI) were calculated for each period, with the baseline period as reference. As a sensitivity analysis, analyses were repeated using only the surveys completed by a family member for the first time.

During the study, 4826 patients were admitted to the ICU and 736 family members (66.3% reference person, 54.3% women) completed 799 questionnaires. Characteristics of the patients and family members were similar across the 4 periods (Additional file 1: Table S3). Among the 23 interventions introduced (mean of 6 per center), 7 (30%), 7 (30%), 5 (22%), 2 (9%), and 2 (9%) were related to the Information, Availability, Comfort, Trust, and Proximity dimensions, respectively (Additional file 1: Table S4). Additional file 1: Fig. S1 shows the distribution of replies for each CCOFS item. The overall proportion of satisfied family members increased significantly over time, from 75.6% at baseline to 87.5% for period 3 (*p* = 0.002, Fig. 1B); the increase concerned four of the dimensions: Comfort (*p* = 0.002), Proximity (*p* = 0.01), Availability (*p* = 0.04), and Support (*p* = 0.04) (Fig. 1C). The odds of relative satisfaction increased from period 1 to period 3, with baseline as reference [OR 1.61 (95% CI 1.04–2.47), 1.89 (1.09–3.29), and 2.23 (1.20–4.15) for periods 1, 2, and 3, respectively]. The improvement in overall satisfaction was consistent when only family members responding to the CCOFS for the first time were considered (*n* = 736) (Additional file 1: Table S5).

One-third of the interventions introduced involved improving information provision. Proactive communication improves families’ understanding of treatment, which is associated with a decreased prevalence of stress symptoms [5]. Consistent with guidelines that call for the ICU environment to be designed to improve the family experience [1], comfort-related interventions, such as improved waiting rooms or signage to orient family members within units, were associated with greater family satisfaction. The CCOFS also identified the need to improve caregiver availability, as previously suggested [3, 4]. Here again, simple and inexpensive measures, such as systematic badge wearing by caregivers, lists of doctors in waiting rooms, or identification of the caregivers in charge at the entrance to each room, helped improve satisfaction.

Our study has several limitations. First, data were obtained from only 15.3% of family members with a possible non-response bias. We cannot exclude that several family members responded to the survey for the same patient; however, two of three respondents were the reference member. Second, the interface may have selected family members who were digitally educated. However, characteristics of the family members were not different from those reported in other similar, non-digital studies [2, 5]. Third, the CCOFS developed for the present study was not validated prior to the study. However, the items and dimensions included are based on recent guidelines and validated scales. Finally, before–after studies are associated with major biases stemming from regression to the mean and the Hawthorne effect.

Implementation of the OpinionFamily program appears to significantly increase overall family satisfaction. Further studies are needed to confirm these results and to evaluate the program’s effectiveness in ultimately improving psychological outcomes of family members.

## Supplementary Information

**Additional file 1**: eAppendix. OpinionFamily Group. **Methods S1**. Scaling and Scoring Methods. **Methods S2**. Real-time analysis and multifaceted improvement interventions. **Table S1**. Critical Care OpinionFamily Survey. **Table S2**. Characteristics of the participating intensive care units. **Table S3**. Patient and family member characteristics for each study period. **Table S4**. Interventions implemented in the four participating intensive care units to improve family satisfaction. **Table S5**. Family satisfaction during each study period using the Critical Care OpinionFamily Survey with only surveys completed by a family member for the first time taken into consideration. **Figure S1**. Distribution of responses from family members for each item of the Critical Care OpinionFamily Survey.

## Data Availability

The trial steering committee (VL and MF) will work to make study data available on legitimate request. Notwithstanding, the steering committee must grant that any proposed publication should be of high quality, and fulfill the related legal and regulatory requirements (e.g., concerning data protection and privacy). The steering committee has the right to review and comment on any draft manuscript prior to publication.

## Abbreviations

CCOFS: Critical Care Opinion Family Survey
CI: Confidence interval
ICU: Intensive care unit
OR: Odds ratios
