# Supplementary material for: Use of the OpinionFamily program to improve satisfaction among families of intensive care unit patients
Source: Crit Care. 2023 May 10;27:181. doi: 10.1186/s13054-023-04445-2 (PMC10173615; doi:10.1186/s13054-023-04445-2)
Supplement: Supplementary file 1 — Additional file 1: eAppendix. OpinionFamily Group. Methods S1. Scaling and Scoring Methods. Methods S2. Real-time analysis and multifaceted improvement interventions. Table S1. Critical Care OpinionFamily Survey. Table S2. Characteristics of the participating intensive care units. Table S3. Patient and family member characteristics for each study period. Table S4. Interventions implemented in the four participating intensive care units to improve family satisfaction. Table S5. Family satisfaction during each study period using the Critical Care OpinionFamily Survey with only surveys completed by a family member for the first time taken into consideration. Figure S1. Distribution of responses from family members for each item of the Critical Care OpinionFamily Survey. [file 13054_2023_4445_MOESM1_ESM.docx]

**Additional file 1**

[eAppendix. OpinionFamily Group 2](#_Toc132056332)

[Methods S1. Scaling and Scoring Methods 3](#_Toc132056333)

[Methods S2. Real-time analysis and multifaceted improvement interventions 4](#_Toc132056334)

[Table S1. Critical Care OpinionFamily Survey 5](#_Toc132056335)

[Table S2. Characteristics of the participating intensive care units 6](#_Toc132056336)

[Table S3. Patient and family member characteristics for each study period 7](#_Toc132056337)

[Table S4. Interventions implemented in the four participating intensive care units to improve family satisfaction 9](#_Toc132056338)

[Table S5. Family satisfaction during each study period using the Critical Care OpinionFamily Survey with only surveys completed by a family member for the first time taken into consideration^a^ 11](#_Toc132056339)

[Figure S1. Distribution of responses from family members for each item^a^ of the Critical Care OpinionFamily Survey (n=799) 12](#_Toc132056340)

# eAppendix. OpinionFamily Group

**Service de Médecine Intensive Réanimation, Département Médico‐Universitaire APPROCHES, Centre Hospitalier Universitaire Tenon, Assistance Publique‐Hôpitaux de Paris, Sorbonne Université, Paris, France**: Vincent Labbé, Muriel Fartoukh, Aude Gibelin, Guillaume Voiriot, Michel Djibré, Clarisse Blayau

**Département Médico‐Universitaire SAPHIRE, Hôpitaux Universitaires Henri Mondor‐Albert Chenevier, Assistance Publique‐Hôpitaux de Paris, Créteil, France**: Irma Bourgeon-Ghittori

**Service de Médecine Intensive Réanimation, Département Médico‐Universitaire Médecine, Hôpitaux Universitaires Henri Mondor‐Albert Chenevier, Assistance Publique‐Hôpitaux de Paris, Université Paris Est Créteil, Créteil, France :** Keyvan Razazi, Armand Mekontso Dessap

**Service de Médecine Intensive Réanimation, Centre Hospitalier Universitaire Pitié‐Salpêtrière, Assistance Publique‐Hôpitaux de Paris, Sorbonne Université, Paris, France**: Elise Morawiec, Martin Dres, Maxens Decavele, Alexandre Demoule

**Service de Médecine Intensive Réanimation, Centre Hospitalier Universitaire Saint‐Antoine, Assistance Publique‐Hôpitaux de Paris, Sorbonne Université, Paris, France**: Naïke Bigé, Hafid Ait-Oufella, Bertrand Guidet, Jérémie Joffre

**Department of Clinical Pharmacology and Clinical Research Platform Paris‐East (URCEST‐CRC‐CRB), Centre Hospitalier Universitaire Saint‐Antoine, Assistance Publique‐Hôpitaux de Paris, Sorbonne Université, Paris, France**: Sophie Tuffet, Alexandra Rousseau

# Methods S1. Scaling and Scoring Methods

The Critical Care OpinionFamily Survey (CCOFS) was available in French, English, and Arabic and all statements were written in the positive tense. For ease of use, the degree of agreement with a given item was recorded by selecting the corresponding stars on a Likert scale [1] (0.5-1 star: strongly disagree, very dissatisfied; 1.5-2 stars: disagree, not satisfied; 2.5-3 stars: neither agree nor disagree, not certain; 3.5-4 stars: agree, satisfied; 4.5-5 stars: strongly agree, very satisfied). Each half-star was equal to 0.5 points; therefore, the possible score for each item ranged from 0.5 (minimum score) to 5 (maximum score) points. The items were presented in random order to each respondent. The score for each dimension was calculated as the mean of all scores for the corresponding items. The overall score was calculated as the mean of all the CCOFS item scores. A score ≥ 3.5 was considered to indicate satisfaction. The satisfaction survey was followed by questions related to the demographic characteristics of the family members (age, sex, relationship to the relative, level of spoken French, number of previous visits, previous response to the CCOFS, and days elapsed since the patient’s admission).

# Methods S2. Real-time analysis and multifaceted improvement interventions

The framework for developing corrective actions and auditing them was a rapid-cycle change methodology based on Plan-Do-Study-Act (PDSA) cycles applied successfully in many health domains [2][3]. The PDSA framework allows for identification of gaps within the organization (Plan), implementation of interventions (Do), monitoring of real-time changes in referrals (Study), and adjustment of interventions as barriers are identified (Act). During the study period, the investigators received the CCOFS report for their center every 3 months: for the first three months (baseline period), for the 4^th^ to 6^th^ month (period 1), for the 7^th^ to 9^th^ month (period 2), and for the 10^th^ to 12^th^ month (period 3). Based on the reports, working groups composed of physicians and nurses from each of the participating centers, were invited to implement improvement interventions without delay, focusing on the items that did not meet satisfaction. The choice of improvement actions was left to the discretion of the working groups and was collected prospectively. To account for different ICU organization, each of periods 1, 2, and 3 (and corresponding reports) started on the day following the analysis of the report by the working groups. The duration of each period for each center is shown in the following Table:

| **Variable** | **mean ± standard deviation** | | | | |
| --- | --- | --- | --- | --- | --- |
|  | **Total** | **Center 1** | **Center 2** | **Center 3** | **Center 4** |
| **Duration of study period, months** |  |  |  |  |  |
| Baseline period | 3.1 ± 0.3 | 3.2 | 3.5 | 2.9 | 2.7 |
| Period 1 | 4.4 ± 0.6 | 3.9 | 5.2 | 4.0 | 4.6 |
| Period 2 | 2.4 ± 0.7 | 2.3 | 1.6 | 3.2 | 2.4 |
| Period 3 | 2.2 ± 0.4 | 2.7 | 1.8 | 2.0 | 2.4 |

# Table S1. Critical Care OpinionFamily Survey

| **Proximity- Family and the patient** |
| --- |
| I01-I feel my privacy with my relative is respected when I visit. |
| I02-I can stay by my relative as long as I want. |
| I03-I feel the period before seeing my relative is reasonable. |
| I04-I can participate in some of the medical care of my relative. |
| **Availability- Family and the caregivers** |
| I05-I think the doctors are available to talk to me on a regular basis. |
| I06-I think the nurses are available to talk to me on a regular basis. |
| I07-I can easily identify the nurse in charge of my relative. |
| I08-I can easily identify the doctor in charge of my relative. |
| **Information- Family and the caregivers** |
| I09-I receive honest information regarding my relative’s health. |
| I10-I understand the reason (s) for which my relative is hospitalized in intensive care. |
| I11-I understand the utility of the treatment and the devices in my relative’s room. |
| I12-I understand the evolution of my relative’s health. |
| **Trust- Family and the caregivers** |
| I13-I feel my relative is getting the best medical care. |
| I14-I feel the medical staff is paying attention to my relative. |
| I15-I feel I will be informed immediately should my relative’s health worsen. |
| I16-I feel the healthcare staff is reactive when the bell rings or if an emergency arises. |
| **Support- Family and the caregivers** |
| I17-I feel the healthcare staff is nice to me. |
| I18-I feel the healthcare staff is sensitive to my needs. |
| I19-I was welcomed nicely by the staff when I arrived in the intensive care unit. |
| I20-I feel comfortable asking questions if I need to. |
| **Comfort- Family and the environment** |
| I21-I appreciate the comfort and the cleanliness of the waiting room. |
| I22-I appreciate the quality, the cleanliness of the facilities and of my relative’s room. |
| I23- I feel comfortable whenever I visit my relative. |
| I24- I feel the noise level within the intensive care unit is reasonable. |

Abbreviations: I, Item

# Table S2. Characteristics of the participating intensive care units

| **Characteristic** | **Total, n=4** |
| --- | --- |
| Number of ICU beds, mean ± SD | 21 ± 3 |
| Number of attending physicians, mean ± SD | 10 ± 2 |
| Nurse/patient ratio, mean ± SD | 2.8 ± 0.2 |
| Number of total daily visits for family members, mean ± SD | 16 ± 9 |
| 24-hour visiting policy, No. (%) | 2 (50) |
| Waiting room (%) | 4 (100) |
| Family meeting room (%) | 4 (100) |
| Family information booklet (%) | 4 (100) |
| Regular nurse-physician meetings about family information (%) | 1 (25) |

# Table S3. Patient and family member characteristics for each study period

|  |  | | | | |
| --- | --- | --- | --- | --- | --- |
| **Variable** | **Total** | **Baseline** | **Period 1** | **Period 2** | **Period 3** |
| **Patient characteristics** | **n=4826** | **n=1217** | **n=1797** | **n=944** | **n=868** |
| Age, mean ± sd, y | 58.4 ± 18.5 | 60.1 ± 18.3 | 57.9 ± 18.9 | 57.5 ± 18.1 | 58.3 ± 18.0 |
| Female, n (%) | 1823 (37.8) | 477 (39.2) | 659 (36.7) | 354 (37.5) | 333 (38.4) |
| SAPSII, mean ± sd | 37.8 ± 22.5 | 37.4 ± 22.9 | 38.6 ± 23.5 | 37.1 ± 20.3 | 37.5 ± 22.0 |
| Admission category |  |  |  |  |  |
| Surgery | 621 (12.9) | 164 (13.5) | 234 (13.0) | 115 (12.2) | 108 (12.4) |
| Medical | 4205 (87.1) | 1053 (86.5) | 1563 (87.0) | 829 (87.8) | 760 (87.6) |
| Mechanical invasive ventilation, n (%) | 1491 (30.9) | 368 (30.2) | 578 (32.2) | 270 (28.6) | 275 (31.7) |
| ICU length of stay, median (IQR), d | 4 (3-7) | 4 (3-7) | 4 (3-7) | 4 (3-7) | 4 (3-8) |
| Death in ICU, n (%) | 567 (11.7) | 138 (11.3) | 215 (12.0) | 105 (11.1) | 109 (12.6) |
| **Family member characteristics** | **N=736** | **n=224** | **n=268** | **n=132** | **n=112** |
| Age, mean ± sd, y |  |  |  |  |  |
| 15-24 | 83 (11.3) | 21 (9.4) | 33 (12.3) | 17 (12.9) | 12 (10.7) |
| 25-34 | 142 (19.3) | 39 (17.4) | 50 (18.7) | 24 (18.2) | 29 (25.9) |
| 35-44 | 162 (22.0) | 54 (24.1) | 60 (22.4) | 30 (22.7) | 18 (16.1) |
| 45-54 | 156 (21.2) | 49 (21.9) | 59 (22.0) | 24 (18.2) | 24 (21.4) |
| 55-64 | 117 (15.9) | 28 (12.5) | 48 (17.9) | 23 (17.4) | 18 (16.1) |
| 65-74 | 63 (8.6) | 25 (11.2) | 17 (6.3) | 12 (9.1) | 9 (8.0) |
| >=75 | 13 (1.8) | 8 (3.6) | 1 (0.4) | 2 (1.5) | 2 (1.8) |
| Female, n (%) | 400 (54.3) | 129 (57.6) | 153 (57.1) | 66 (50.0) | 52 (46.4) |
| Level of spoken French, n (%) |  |  |  |  |  |
| Perfect | 689 (93.6) | 208 (92.9) | 253 (94.4) | 126 (95.5) | 102 (91.1) |
| Good | 27 (3.7) | 6 (2.7) | 8 (3.0) | 4 (3.0) | 9 (8.0) |
| Average | 11 (1.5) | 4 (1.8) | 5 (1.9) | 1 (0.8) | 1 (0.9) |
| Low | 4 (0.5) | 3 (1.3) | 1 (0.4) | 0 (0) | 0 (0) |
| Not at all | 5 (0.7) | 3 (1.3) | 1 (0.4) | 1 (0.8) | 0 (0) |
| Reference person, n (%) | 488 (66.3) | 139 (62.1) | 184 (68.7) | 88 (66.7) | 77 (68.8) |
| Relationship to the patient |  |  |  |  |  |
| Husband/wife/partner | 103 (14.0) | 30 (13.4) | 34 (12.7) | 25 (18.9) | 14 (12.5) |
| Companion | 42 (5.7) | 10 (4.5) | 20 (7.5) | 6 (4.5) | 6 (5.4) |
| Child | 240 (32.6) | 91 (40.6) | 72 (26.9) | 46 (34.8) | 31 (27.7) |
| Parent | 105 (14.3) | 24 (10.7) | 43 (16.0) | 16 (12.1) | 22 (19.6) |
| Sibling | 89 (12.1) | 25 (11.2) | 42 (15.7) | 12 (9.1) | 10 (8.9) |
| Other family member | 111 (15.1) | 31 (13.8) | 44 (16.4) | 17 (12.9) | 19 (17.0) |
| No family relation | 46 (6.3) | 13 (5.8) | 13 (4.9) | 10 (7.6) | 10 (8.9) |
| Length of stay of the patient in the ICU in days, n (%)^a^ |  |  |  |  |  |
| 1 | 73 (9.9) | 35 (15.6) | 19 (7.1) | 15 (11.4) | 4 (3.6) |
| 2 | 112 (15.2) | 31 (13.8) | 43 (16.0) | 19 (14.4) | 19 (17.0) |
| 3 | 102 (13.9) | 27 (12.1) | 44 (16.4) | 17 (12.9) | 14 (12.5) |
| 4-7 | 213 (28.9) | 57 (25.4) | 86 (32.1) | 36 (27.3) | 34 (30.4) |
| 8-14 | 127 (17.3) | 42 (18.8) | 43 (16.0) | 20 (15.2) | 22 (19.6) |
| 15-28 | 65 (8.8) | 23 (10.3) | 16 (6.0) | 12 (9.1) | 14 (12.5) |
| > 28 | 44 (6.0) | 9 (4.0) | 17 (6.3) | 13 (9.8) | 5 (4.5) |
| Number of previous visits to the ICU ^a^, n (%) |  |  |  |  |  |
| 1-3 | 305 (41.4) | 95 (42.4) | 115 (42.9) | 51 (38.6) | 44 (39.3) |
| 4-10 | 247 (33.6) | 79 (35.3) | 90 (33.6) | 37 (28.0) | 41 (36.6) |
| >10 | 184 (25.0) | 50 (22.3) | 63 (23.5) | 44 (33.3) | 27 (24.1) |

Abbreviations: ICU, intensive care unit; IQR, interquartile; SAPS, Simplified Acute Physiology Score; SD, standard deviation

^a^ On the day when the family member responded for the first time (one family member could complete several surveys at different times during their relative's stay in the intensive care unit)

# Table S4. Interventions implemented in the four participating intensive care units to improve family satisfaction

| **Center** | **Implementation period ^a^** | **Dimension ^b^** | **Description ^b^** |
| --- | --- | --- | --- |
| 1 | 1 | Comfort | Wall decoration in the waiting room. |
| 1 | 1 | Comfort | Signage on the walls and floor (colored strips) to orientate family members within the units. |
| 1 | 2 | Trust | Systematic double verification that the reference person’s telephone number is registered in the medical record. |
| 1 | 3 | Information | Educational films shown in the waiting room on the main procedures and equipment used in the intensive care unit. |
| 2 | 1 | Comfort | Change of furniture in the waiting room; daily check of waiting room cleanliness by the caregivers; note to relatives on the need to keep the waiting room clean. |
| 2 | 1 | Availability | List of doctors with photographs in the waiting room; requirement for caregivers to introduce themselves to relatives with name and function; names of doctors in charge of patients indicated at the entrance of each unit. |
| 2 | 2 | Availability | Systematic badge for all caregivers. |
| 2 | 2 | Availability  Information | Structured and scheduled meetings between doctors and relatives. |
| 3 | 1 | Availability | Directory of doctors with photographs in the waiting room. |
| 3 | 1 | Availability | Names of nurses in charge of patients indicated at the entrance of each chamber. |
| 3 | 1 | Information | Establishment of a procedure regarding information given to families. |
| 3 | 2 | Comfort | Decrease the intensity levels of the alarms and the improve differentiation of day/night light sources. |
| 3 | 2 | Proximity | Implement dedicated telephones to communicate between the waiting room and the heath care stations. |
| 3 | 3 | Comfort | Wall decoration change of furniture in the waiting room; daily check of the waiting room cleanliness by the caregivers; note to relatives on the need to keep the waiting room clean. |
| 4 | 1 | Availability | Names of the nurse, the doctor and the fellow in charge of the patient indicated at the entrance of each chamber. |
| 4 | 1 | Trust | Introduce the following point into the first structured meeting with the relative: list of events requiring immediate notification to the relative (ie, intubation, emergency surgery, emergency transfer to another hospital). |
| 4 | 1 | Availability  Information | Make a systematic appointment with the doctor at the end of each structured meeting. Date of next appointment posted in the patient's room. |
| 4 | 1 | Information | Directory of doctors with photographs in the waiting room. |
| 4 | 1 | Information | Improve the nurse-physician liaison regarding the content of information given to relatives in order to avoid contradictory information: oral communication with dedicated meetings and written communication using medical records. |
| 4 | 2 | Proximity | Screen in the waiting room to indicate the presence of caregivers in the patient's room. |
| 4 | 2 | Information | Structured meeting at ICU admission. |
| 4 | 2 | Information | Limit information provided by phone to avoid misunderstandings or projections. |
| 4 | 2 | Information | Structured and formal meeting with physicians and nurses. |

^a^ after baseline period.

^b^ collected prospectively by principal investigators

# Table S5. Family satisfaction during each study period using the Critical Care OpinionFamily Survey with only surveys completed by a family member for the first time taken into consideration^a^

|  | No./total No (%) | | | | *P* –value ^b^ |
| --- | --- | --- | --- | --- | --- |
| Variable | Baseline n=224 | Period 1 n=268 | Period 2 n=132 | Period 3 n=112 |  |
| Overall | 178 (79.5) | 225 (84.0) | 115 (87.1) | 97 (86.6) | 0.04 |
| Proximity to the patient | 164 (73.2) | 195 (72.8) | 106 (80.3) | 86 (76.8) | 0.21 |
| Comfort | 175 (78.1) | 208 (77.6) | 111 (84.1) | 97 (86.6) | 0.03 |
| Family and caregivers |  |  |  |  |  |
| Availability | 162 (66.9) | 209 (72.8) | 117 (78.0) | 89 (74.2) | 0.21 |
| Information | 197 (81.4) | 251 (87.5) | 127 (84.7) | 105 (87.5) | 0.54 |
| Trust | 201 (83.1) | 252 (87.8) | 128 (85.3) | 103 (85.8) | 0.80 |
| Support | 197 (81.4) | 239 (83.3) | 131 (87.3) | 106 (88.3) | 0.29 |

^a^ Each family member could complete the survey several times during their relative's stay in the intensive care unit

^b^ Mantel-Haenszel’s Chi square test for trend

# Figure S1. Distribution of responses from family members for each item^a^ of the Critical Care OpinionFamily Survey (n=799)

**
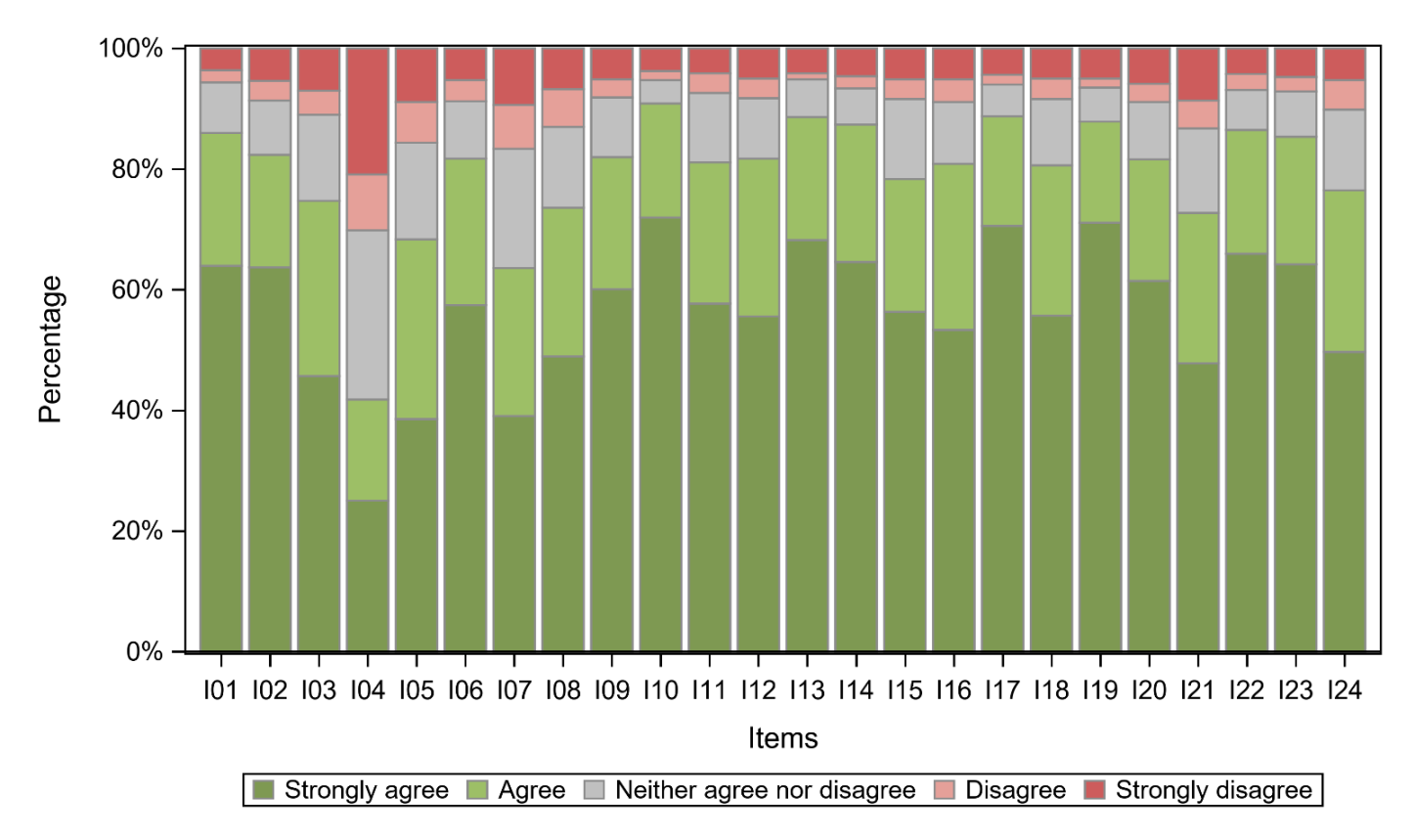
**

Abbreviation: I, Item

^a^ I01 to I24: see Table S2 for explanation

**References**

1. Likert R, Roslow S, Murphy G. A simple and reliable method for scoring the Thurstone attitude sclale. Pers Psychol. 1993;46:689–90.

2. Yang Z, Ma X, Chen Y, Cao Y, Li Q, Pan X, et al. Effects of a Quality Improvement Program to Reduce Central Venous Catheter-Related Infections in Hemodialysis Patients. Am J Med Sci. 2021;361:461–8.

3. Taylor MJ, McNicholas C, Nicolay C, Darzi A, Bell D, Reed JE. Systematic review of the application of the plan-do-study-act method to improve quality in healthcare. BMJ Qual Saf. 2014;23:290–8.
